# miR-3065-3p promotes stemness and metastasis by targeting CRLF1 in colorectal cancer

**DOI:** 10.1186/s12967-021-03102-y

**Published:** 2021-10-16

**Authors:** Yifan Li, Jing Xun, Botao Wang, Yuan Ma, Lanqiu Zhang, Lei Yang, Ruifang Gao, Jun Guan, Tianyu Liu, Hejun Gao, Ximo Wang, Qi Zhang

**Affiliations:** 1 Academy of Medical Engineering and Translational Medicine, Tianjin University, Tianjin, China; 2 Tianjin Key Laboratory of Acute Abdomen Disease Associated Organ Injury and Integrated Chinese and Western Medicine (ITCWM) Repair, Institute of Integrative Medicine for Acute Abdominal Diseases, Integrated Chinese and Western Medicine Hospital, Tianjin University, Tianjin, China; 3 Tianjin Key Laboratory of Acute Abdomen Disease Associated Organ Injury and Integrated Chinese and Western Medicine (ITCWM) Repair, Graduate School of Tianjin Medical University, Tianjin, China; 4 Tianjin Institute of Medical and Pharmaceutical Sciences, Tianjin, China

**Keywords:** miR-3065-3p, CRLF1, Stemness, Metastasis, Colorectal cancer

## Abstract

**Background:**

Colorectal cancer is one of the most common malignancy in the world. It has been reported that cancer stem cells (CSCs) serve as the primary drivers of tumorigenesis and tumor progression. There is an urgent need to explore novel molecules that regulate CSCs or their signatures. Increasing evidence has shown that miRNAs are involved in tumorigenesis and progression. Here, we aim to explore the regulatory effect and mechanism of miR-3065-3p on the stemness of colorectal cancer.

**Methods:**

The expression of miR-3065-3p in colorectal cancer and the association of miR-3065-3p expression with prognosis of patients with colorectal cancer were analyzed using TCGA dataset or clinical cases. Gain or loss of function in different models, including colorectal cancer cell lines and orthotopic xenograft or liver metastatic mouse model, were used to investigate the effects of miR-3065-3p on colorectal cancer stemness and metastasis in vitro and in vivo. Cancer stemness was analyzed by detecting the ability of migration and invasion, NANOG, OCT4, and SOX2 expression, ALDH activity and sphere formation. In addition, the interaction of miR-3065-3p and cytokine receptor-like factor 1 (CRLF1) was analyzed theoretically and identified by the luciferase reporter assay. Moreover, the correlation between CRLF1 expression and miR-3065-3p was analyzed in colorectal cancer tissues. Finally, the effect of CRLF1 on the stemness and metastasis of colorectal cancer in vitro and in vivo was assessed.

**Results:**

In this report, we found that miR-3065-3p was overexpressed in colorectal cancer and that its high expression was associated with poor prognosis of patients with colorectal cancer. miR-3065-3p promotes the stemness and metastasis of colorectal cancer. Furthermore, CRLF1 was the downstream target of miR-3065-3p and inhibited the stemness of colorectal cancer. In addition, CRLF1 expression was negatively correlated with miR-3065-3p in colorectal cancer tissues. And, CRLF1 mediated the effects of miR-3065-3p on promoting stemness of colorectal cancer cells.

**Conclusion:**

Our data suggest that miR-3065-3p promoted the stemness and metastasis of colorectal cancer by targeting CRLF1. miR-3065-3p might serve as a promising prognostic marker as well as a therapeutic target for colorectal cancer.

**Supplementary Information:**

The online version contains supplementary material available at 10.1186/s12967-021-03102-y.

## Background

Colorectal cancer (CRC) is one of the most common primary malignancies of the digestive tract [1].Although treatment strategies for colorectal cancer, including surgery, chemotherapy, radiotherapy, and immunotherapy, have been improved, colorectal cancer still ranks third in morbidity and mortality among malignancies worldwide [2, 3]. There is an urgent need to explore its pathogenesis and discover effective therapies for colorectal cancer.

Cancer stem cells (CSCs), a small subset of cancer cells, possess self-renewal properties and the ability to differentiate into multiple cell types [4]. The key property of CSCs is an exclusive capacity for tumorigenesis. In addition, CSCs serve as primary drivers of cancer recurrence or relapse [5]. Although several markers, including aldehyde dehydrogenase (ALDH), have been utilized to identify and investigate CSCs [6–8], most of the current therapies for CRC do not show ideal effects against CSCs. Thus, there is a demand for the identification of novel molecules that regulate CSCs.

miRNAs are evolutionarily conserved noncoding RNAs composed of 18–25 nucleotides. It has been confirmed that miRNAs function as posttranscriptional repressors of gene expression by targeting the 3ʹ-untranslated regions (UTRs) of target mRNAs and are involved in gene expression pattern fine-tuning, cell differentiation and cell fate determination [9, 10]. Increasing evidence has revealed that miRNAs are key molecules that regulate CSCs during tumor progression [11–13]. Circulating miRNAs have the potential to serve as diagnostic markers of tumor progression and this has been the focus of an increasing number of studies [14, 15]. For example, serum exosomal miR-1247-3p levels in patients with primary hepatic cancer are associated with the extent of lung metastasis [16]. Here, we show that miR-3065-3p functions as an oncogene and promotes the stem cell-like properties of colorectal cancer cells.

It has been reported that miR-3065-3p is involved in tumor progression. miR-3065-3p is repressed in cancer with p53 mutation and its downregulation associated with outcomes of breast cancer and hepatocellular cancer [17]. miR-3065 is associated with LINC01133 and targets retinol metabolism related gene *ADH7* to regulate cervical cancer progression in different age groups [18]. In addition, miR-3065 is associated with altered gene expression regulation in breast cancer [19]. miR-3065 is identified a novel promising candidate in regulating clear cell renal cell carcinogenesis progression by targeting *NRP2* and *FLT1* [20]*.* However, the signature of miR-3065-3p in colorectal cancer has not been investigated and its functional role in modulating development and progression of colorectal cancer has not been elucidated.

In this study, we identified that miR-3065-3p was overexpressed in colorectal cancer and its high expression was associated with poor prognosis of patients with colorectal cancer. Furthermore, we demonstrated that miR-3065-3p promoted the stemness and metastasis of colorectal cancer by targeting Cytokine Receptor Like Factor 1 (CRLF1) in vitro and in vivo. Thus, the data implicates miR-3065-3p as a prognostic marker as well as a therapeutic target for colorectal cancer.

## Materials and methods

### Tumor specimens

Colorectal cancer tissues and adjacent normal tissues from the same patients were collected from the Endoscopy Centre, Tianjin Nankai Hospital. This study was approved by the Clinical Trial Ethics Committee of Tianjin Nankai Hospital (Approval No: NKYY_YX_IRB_2018_039_01), and informed consent was obtained from all patients before their enrollment. All tissues (17 cases) were subjected to histology and pathology, and all samples were immediately frozen in liquid nitrogen at the time of surgery and stored at − 80 °C before use.

### Cell culture

The human CRC cell lines HCT116 and RKO and HEK293T were obtained from the American Type Culture Collection (Manassas, VA, USA) and cultured in high-glucose Dulbecco’s modified Eagle’s medium (DMEM; Invitrogen, Carlsbad, CA, USA) supplemented with 10% fetal bovine serum (FBS; Gibco, USA), streptomycin (100 μg/mL) and penicillin (100 μg/mL). The cells were maintained at 37 °C in a humidified atmosphere of 5% CO_2_.

### Vector construction and establishment of stable cell lines

Human colorectal cancer HCT116 and RKO cell lines stably expressing miR-3065-3p (HCT116-miR-3065-3p and RKO-miR-3065-3p) or negative control (HCT116-NC and RKO-NC), HCT116 cell lines stably expressing CRLF1 (HCT116-CRLF1) or vector control (HCT116-MCS) were established by using a lentivirus transfection system according to the manufacture’s instruction (Biosettia, San Diego, CA, USA).

### Transfection assay

HCT116 and RKO human colorectal cancer cells were transfected with miR-3065-3p mimics or inhibitor using Lipofectamine 3000 Transfection Reagent (Invitrogen) according to the manufacturer’s instructions. The cells were collected for 48 h after transfection. miR-3065-3p mimics or inhibitor, negative control (NC) miRNA and inhibitor negative control (I.NC) were synthesized by RiboBio (Guangzhou, China).

### Real-time quantitative PCR (RT-qPCR)

Total RNA was extracted from the cultured cells and tissues using TRIzol reagent (Invitrogen), and reverse transcription was performed using the TransScript First-Strand cDNA Synthesis SuperMix Kit (TransGen Biotech, Beijing, China) according to the manufacturer’s recommendations. RT-qPCR was performed using an ABI 7500 Fast Real-Time PCR System (Applied Biosystems Thermo Fisher). U6 small nuclear RNA (snRNA) was used as an internal control for miR-3065-3p, and the mRNA levels were normalized to those of GAPDH. The relative gene levels normalized to the control were calculated using the equation 2^−ΔΔCT^, ΔCT = CT_gene_ − CT_U6/GAPDH_, ΔΔCT = ΔCT_test_ − CT_ctrl_. Primers were designed and synthesized, and the sequences are as follow: hsa-miR-3065-3p, forward: 5ʹCGTCAGCACCAGGATATTG3ʹ and reverse: 5ʹGTGCAGGGTCCGAGGT3ʹ; hsa-U6, forward: 5ʹCTCGCTTCGGCAGCACATATACT3ʹ and reverse: 5ʹACGCTTCACGAATTTGCGTGTC3ʹ; homo-NANOG, forward: 5ʹTCTGGACACTGGCTGAATCCT3ʹ and reverse: 5ʹCGCTGATTAGGCTCCAACCAT3ʹ; homo-OCT4, forward: 5ʹGCTCGAGAAGGATGTGGTCC3ʹ and reverse: 5ʹCGTTGTGCATAGTCGCTGCT3ʹ; homo-SOX2, forward: 5ʹGCCTGGGCGCCGAGTGGA3ʹ and reverse: 5ʹGGGCGAGCCGTTCATGTAGGTCTG3ʹ; homo-TMEM47, forward: 5ʹTTGGACATCTGGCACTGCGAGT3ʹ and reverse: 5ʹCCTTCGAGATCCCACGCAGATA3ʹ; homo-CRLF1, forward: 5ʹCCCAGAGAAACCCGTCAACAT3ʹ and reverse: 5ʹACTGTGTGGTACTCCTCACAT3ʹ; homo-CLDN11, forward: 5ʹGGCTGGTGTTTTGCTCATTCTGC3ʹ and reverse: 5ʹAGCACCAATCCAGCCTGCATAC3ʹ; homo-GAPDH, forward: 5ʹCTCTGATTTGGTCGTATTGGG3ʹ and reverse: 5ʹTGGAAGATGGTGATGGGATT3ʹ.

### Western blot

RIPA lysis buffer (Sigma, St. Louis, MO, USA) including protease inhibitor cocktail (Roche, Mannheim, Germany) was used to lyse cells and tissues for total protein extraction. The concentrations of the extract proteins were measured by the BCA kit (SolarBio, Beijing, China). Protein lysates were electrophoresed on 10% SDS-PAGE and transferred onto polyvinylidene fluoride membranes (Millipore, Billerica, MA, USA). The immunoblots were blocked with 5% fat-free dried milk in TBST at room temperature for 1 h and incubated at 4 °C overnight with primary antibodies against NANOG (ab80892, 1:1000, Abcam, Cambridge, MA, USA), OCT4 (ab19857, 1:1000), SOX2 (ab97959, abcam, 1:1000), CRLF1(ab211438, abcam, 1:1000), and β-Actin (ab8226, abcam, 1:5000). The membranes were subsequently incubated with HRP-linked secondary antibodies (ZB-2301 or ZE2305, 1:5000; ZSGB-BIO, Beijing, China). Bound antibodies were detected with enhanced chemiluminescence reagent (Millipore).

### Transwell migration assay

A total of 1 × 10^5^ cells were suspended in serum-free medium and seeded into upper Transwell chambers (8 μm pore size, 24-well plate). The bottom chamber was filled with 500 μL of medium containing 10% FBS. After incubation for 24 h, the inserts were fixed with 4% paraformaldehyde (PFA) for 20 min at room temperature and stained with 0.1% crystal violet staining solution. Nonmigratory cells were removed from the upper chamber with cotton swabs, and migrated cells were visualized using an Olympus microscope (Olympus Co., Tokyo, Japan) and quantified in five fields per chamber under blinded conditions [21–23].

### Wound healing assay

A total of 1 × 10^6^ cells in 2 mL DMEM containing 10% FBS were seeded in each well of a 6-well plate. After the cells were incubated for 24 h, a “wound” was generated by scratching the cells vertically with a 10 μL pipette tip. The floating cells were gently removed with PBS and then cultured with DMEM containing 2% FBS. The wound healing process was assessed at 0 h, 24 h and 48 h using an Olympus microscope (Olympus Co., Tokyo, Japan). The wound healing area was measured in ImageJ software.

### Sphere formation assay

A total of 1 × 10^3^ cells were seeded in ultralow attachment 6-well plates in 3 mL serum-free DMEM supplemented with 1 × B27 (1:50, Invitrogen), 20 ng/mL human epidermal growth factor (Invitrogen), and 20 ng/mL basic fibroblast growth factor (Invitrogen). Spheres larger than 50 μm in diameter were counted after 12 days [12, 24].

### Aldefluor assay

An ALDEFLUOR™ Kit was purchased from Stemcell Technologies. A total of 5 × 10^5^ cells were seeded in 6-well plates and then transiently transfected with miR-3065-3p inhibitor or CRLF1 plasmid. The cells were collected after transfection for 48 h, and the Aldefluor assay was performed according to the manufacturer’s instructions. Stained cells were analyzed on a FACS flow cytometer (Beckman Coulter, Inc.). DEAB, a specific ALDH inhibitor, served as a negative control.

### Dual-luciferase reporter assay

The CRLF1 3ʹUTR containing a predicted miR-3065-3p-targeting seed region or corresponding mutant seed region was inserted into the pmirGLO reporter vector (Promega, Madison, WI, USA) to obtain the pmirGLO-CRLF1 3ʹUTR-WT and the pmirGLO-CRLF1 3ʹUTR-Mut plasmid. To determine the direct targeting relationship between miR-3065-3p and the CRLF1 3ʹUTR, HEK293T cells were cotransfected with miR-3065-3p mimics or scramble negative control (NC) and the wild-type, mutant or empty reporter plasmid [12, 25]. Luciferase activity was measured 40 h later and normalized to Renilla luciferase activity using a Dual-Luciferase Reporter Assay System (Promega) according to the manufacturer’s protocol.

### Animal study

To establish a tumor xenograft mouse model, twenty in total 6-week-old female BALB/c nude mice were randomly divided into four groups with five in each group. HCT116 cells (1.5 × 10^7^) expressing negative control (NC), miR-3065-3p, vector control (MCS) or CRLF1 were subcutaneously injected into the dorsal flanks of mice. Tumor volume was measured twice a week and calculated using the standard formula: length × width^2^/2. The mice were sacrificed one month after injection, and tumor tissues were obtained for further analysis. To construct a mouse model of liver metastasis, cells (3 × 10^6^) were injected into the spleens of mice (five in each group). Liver tissues were obtained 4 weeks after injection, fixed with 4% PFA and stained with hematoxylin and eosin. All animal experiments were performed strictly according to the guidelines for laboratory animals of Tianjin Nankai Hospital and approved by the Institutional Ethics Committees of Tianjin Nankai Hospital (Approval No: NKYY-DWLL-2020-001).

### Hematoxylin–eosin (HE) and immunohistochemical (IHC) staining

Tumors and livers tissues were fixed in 4% PFA, embedded in paraffin, sectioned, and then stained. H&E-stained sections were independently evaluated at 40× and 100× magnification by two pathologists who were blinded to the experimental conditions. IHC-stained sections were processed with an SP Kit (Ovitalin-Biotin Detection System for Streptomyces Rabbits) purchased from ZSGB-BIO according to the manufacturer’s protocol. Positive signals were detected by using DAB color developing agent. Images were captured using a Leica microscope. The protein expression level was determined by measuring the mean densitometry and assessed with ImageJ software. By measuring the integrated optical density (IOD) and area of each image, the average optical density (mean density = IOD/area) was calculated, which reflects the per unit area concentration of the target protein. The signal density of the tissue areas was measured in at least three sections.

### Bioinformatics tools

RNA-seq data of colorectal cancer patients from TCGA database using the UALCAN (http://ualcan.path.uab.edu/) were used for miR-3065-3p or CRLF1 expression analysis [26]. Kaplan–Meier analysis was performed using the OncomiR tool (http://www.oncomir.org/) [27]. miRNA target prediction and analysis were performed using algorithms from TargetScan (http://www.targetscan.org/) [28], miRDB (http://www.mirdb.org/) [29], and GEPIA (http://gepia.cancer-pku.cn/) [30].

### Statistical analysis

All data shown are representative of at least three independent experiments and are presented as the mean ± SEM. Student’s t-test or nonparametric test was used to compare two independent groups and paired groups. One-way ANOVA followed by the Tukey–Kramer multiple comparisons test was performed to compare three or more groups within the same experiment. Pearson correlation coefficients were used to assess the degree of association between biomarkers. All statistical analyses were computed and are presented via GraphPad Prism 8 software. The results were considered statistically significant when *P < 0.05, **P < 0.01, and ***P < 0.001.

## Results

### miR-3065-3p is highly expressed and its expression correlates with patient survival in colorectal cancer

**Fig. 1:**
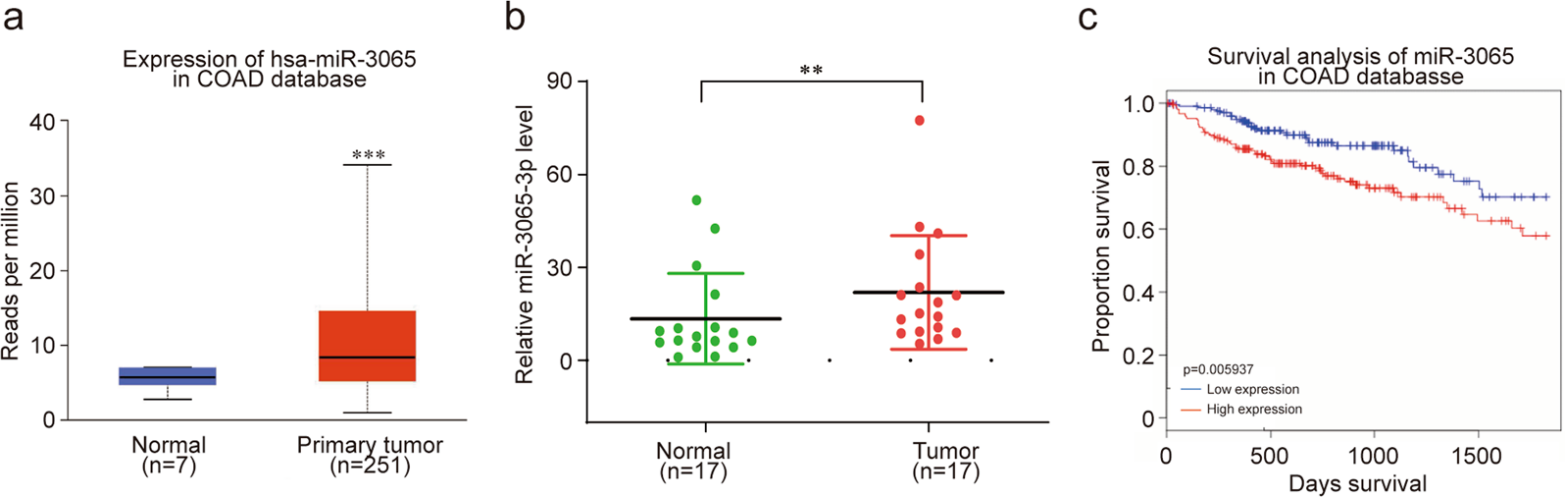
miR-3065-3p is highly expressed and its expression correlates with patient survival in colorectal cancer. **a** Analysis of miR-3065-3p expression in colorectal cancer and normal tissues according to data from TCGA database (analysis generated from UALCAN: http://ualcan.path.uab.edu/index.html). **b** RT-qPCR analysis of miR-3065-3p expression in 17 pairs of adjacent normal and colorectal cancer tissues. **c** Kaplan–Meier analysis showing the overall survival (OS) curves of CRC patients with different expression of miR-3065-3p. The data shown represent the mean values ± SEM of three independent experiments. *P < 0.05; **P < 0.01; ***P < 0.001

To evaluate the expression of miR-3065-3p in colorectal cancer, we analyzed TCGA database using the UALCAN website (http://ualcan.path.uab.edu/). The results showed that miR-3065-3p expression was significantly upregulated in CRC tissues compared to normal tissues (Fig. 1a). To confirm the above-mentioned results, we measured the expression of miR-3065-3p in samples from colorectal cancer patients, which included 17 pairs of adjacent normal and colorectal cancer tissues. The results confirmed that the expression of miR-3065-3p was increased in human colorectal cancer tissues (Fig. 1b). More importantly, Kaplan–Meier analysis was performed using the OncomiR database (http://www.oncomir.org). Briefly, the expression of miR-3065-3p was ranked from low to high and the median was taken as the cut-off. The results showed that the overall survival rate of colorectal cancer patients with high miR-3065-3p expression was poorer than that of patients with low miR-3065-3p expression (Fig. 1c). These data indicated that the expression of miR-3065-3p correlates with poor outcome in CRC patients.

### miR-3065-3p promotes the stemness of colorectal cancer cells in vitro

**Fig. 2:**
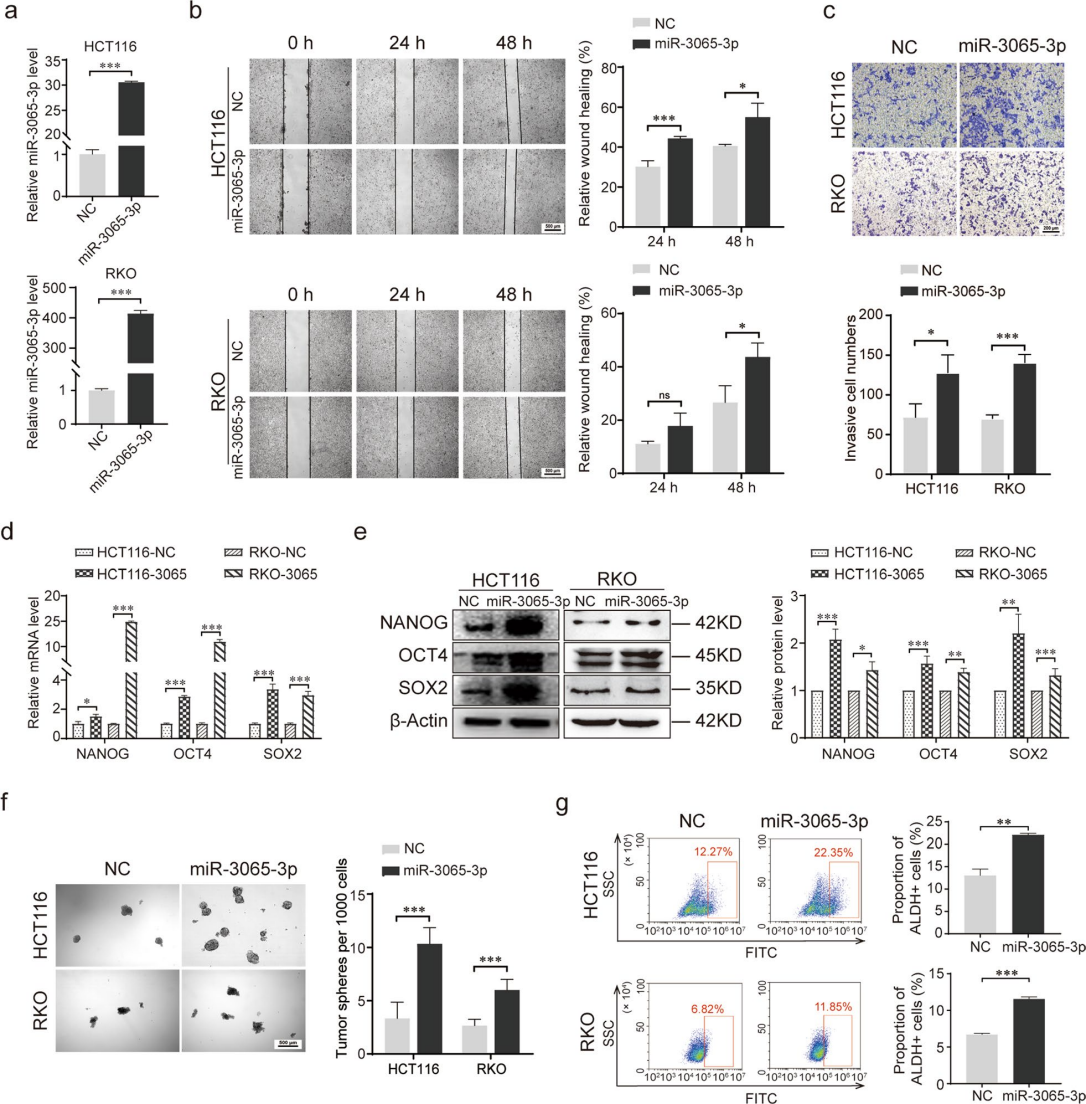
miR-3065-3p promotes the stemness of colorectal cancer cells in vitro. **a** RT-qPCR analysis of miR-3065-3p overexpression in HCT116 and RKO cells. **b** Representative images of the wound healing assay and statistical results of the migration area are shown. **c** Transwell assay of the migration of miR-3065-3p-overexpressing HCT116 or RKO cells compared and negative control (NC)-expressing cells and the statistical results. **d**, **e** The mRNA (**d**) and protein (**e**) levels of NANOG, OCT4, and SOX2 in miR-3065-3p-overexpressing HCT116 or RKO cells were analyzed by RT-qPCR and western blotting. **f** Representative images and statistical results of sphere formation assays. Statistical results are shown in the right panel. **g** Flow cytometric analysis of ALDH activity in HCT116 and RKO cells transfected with miR-3065-3p mimics or negative control (NC). Statistical results are shown in the right panel. The data shown represent the mean values ± SEM of three independent experiments. *P < 0.05; **P < 0.01; ***P < 0.001

We next explored the role of miR-3065-3p in the metastasis and stemness of colorectal cancer cells in vitro. Stable overexpression of miR-3065-3p in human CRC cell lines HCT116 and RKO was established via a lentivirus system (Fig. 2a). Wound healing and Transwell assays showed that overexpression of miR-3065-3p significantly enhanced both the invasion and migration capacity of CRC cells (Fig. 2b, c). Then, stem cell-like characteristics were investigated. Our results showed that the overexpression of miR-3065-3p increased the mRNA and protein expression levels of the stem cell-related transcription factors NANOG, OCT4 and SOX2 (Fig. 2d, e). Ectopic miR-3065-3p expression also promoted the sphere formation ability of CRC cells (Fig. 2f). In addition, flow cytometry assays demonstrated that overexpression of miR-3065-3p increased the percentage of ALDEFLUOR + cells among HCT116 and RKO cells (Fig. 2g). Consistent with miR-3065-3p overexpression, inhibition of miR-3065-3p suppressed the stem cell-like characteristics, including migration and invasion ability, ALDH activity and stemness-related transcription factors expression, in colorectal cancer cells (Additional file 1: Figure S1). Taken together, these results demonstrated that miR-3065-3p promotes the stemness and metastasis of colorectal cancer in vitro.

### miR-3065-3p promotes the tumorigenesis and metastasis of colorectal cancer in vivo

**Fig. 3:**
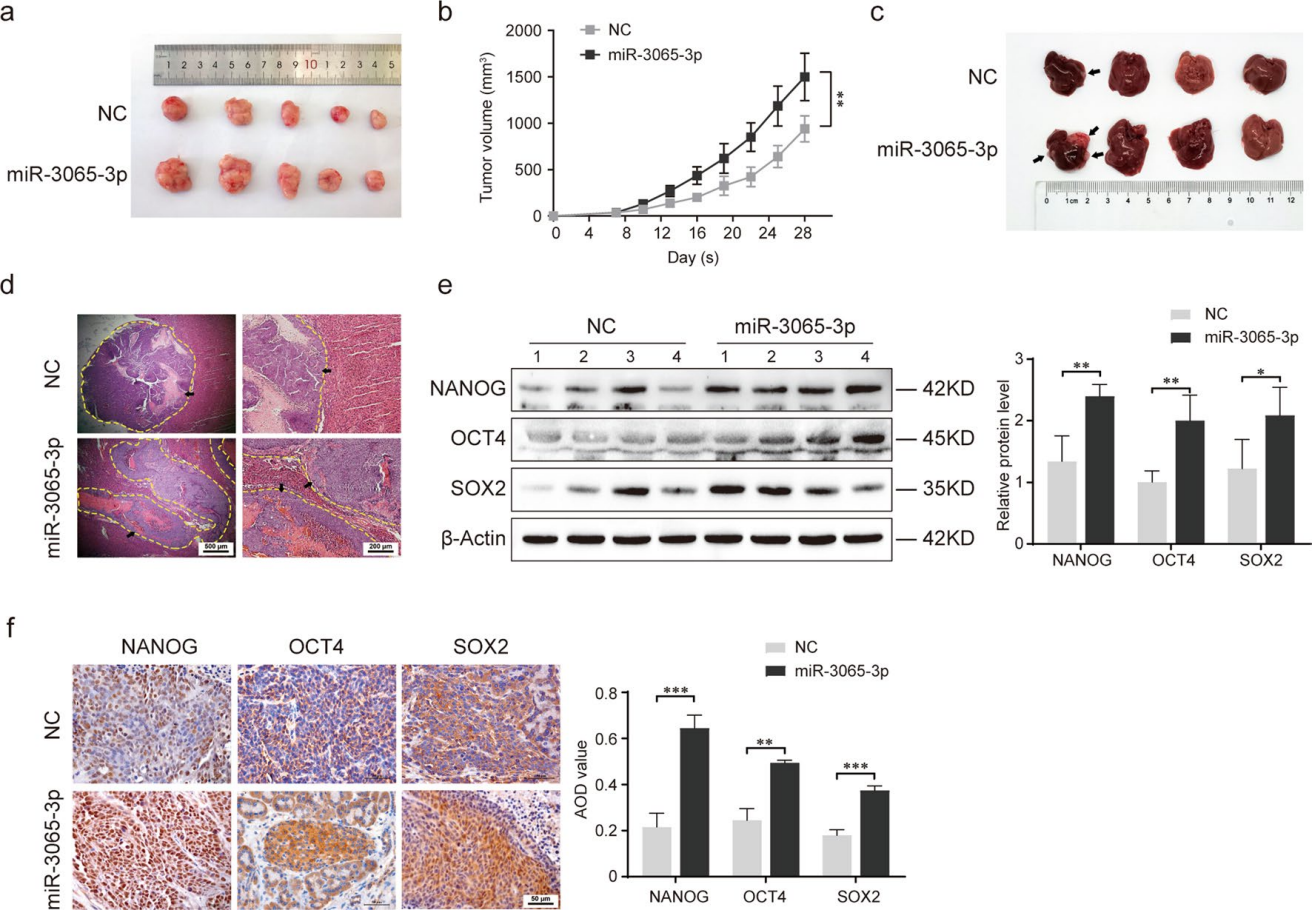
miR-3065-3p promotes the tumorigenesis and metastasis of colorectal cancer in vivo. **a**, **b** A mouse model of colorectal cancer with five mice in each group was established via orthotopic implantation of HCT116 cells overexpressing miR-3065-3p or negative control (NC). Images showing tumor size (**a**) and statistical results of tumor volume (**b**) are shown. A liver metastasis mouse model of colorectal cancer with five mice in each group was established via spleen injection of miR-3065-3p-overexpressing HCT116 or negative control (NC) cells. **c**, **d** Metastatic nodules of the liver (**c**) and representative images of HE-stained metastatic lesions (**d**) are shown. **e**,** f** Western blot (**e**) and immunohistochemical (**f**) analysis of NANOG, OCT4, and SOX2 expression in tumor tissue from xenograft mice. ‘1–4’ represented the different four mice in the same group. Statistical results are shown in the right panel. The data shown represent the mean values ± SEM of three independent experiments. *P < 0.05; **P < 0.01; ***P < 0.001

To further confirm the above findings, orthotopic xenograft and liver metastasis mouse models of colorectal cancer were established in vivo via subcutaneous injection and spleen injection of miR-3065-3p-overexpressing HCT116 or control cells, respectively. We observed that HCT116 cells overexpressing miR-3065-3p resulted in a significantly larger tumor volume than controls cells in the xenograft model (Fig. 3a, b). Moreover, number of metastatic foci in the liver was increased in mice ectopically expressing miR-3065-3p compared with control mice (Fig. 3c, d). Furthermore, we determined the expression of stemness-related markers in tumor tissues by western blot analysis and immunohistochemistry. The results showed that miR-3065-3p overexpression in HCT116 cells led to an increase in the expression of the stem cell markers NANOG, OCT4 and SOX2 in tumor tissue (Fig. 3e, f). Taken together, these findings indicated that miR-3065-3p facilitates the tumorigenesis and metastasis of colorectal cancer.

### CRLF1 is a downstream target of miR-3065-3p in colorectal cancer cells

**Fig. 4:**
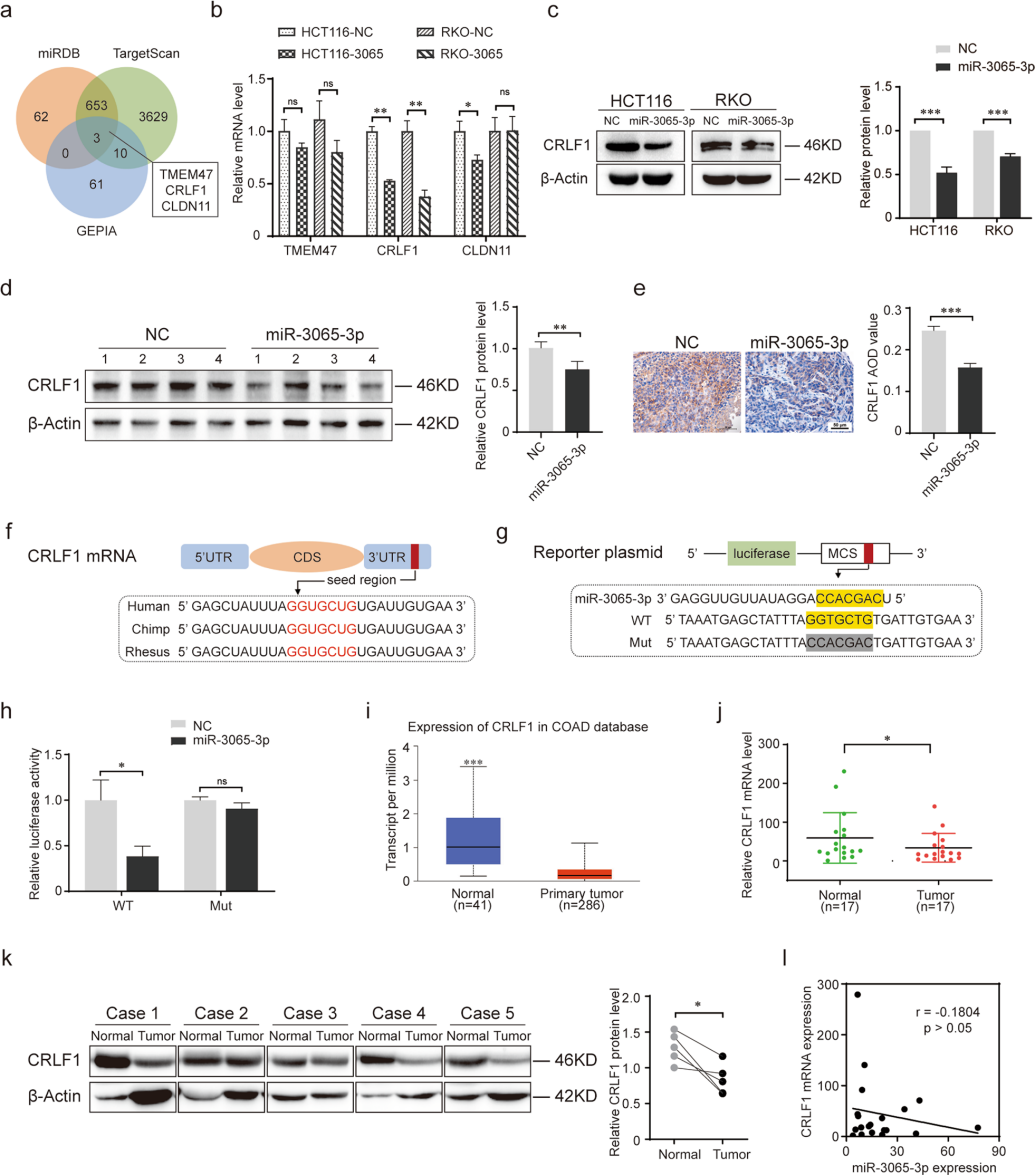
CRLF1 is a downstream target of miR-3065-3p in colorectal cancer cells. **a** Venn diagrams showing the number of genes identified as potential targets of miR-3065-3p using three databases. **b** RT-qPCR analysis of TMEM47, CRLF1 and CLDN11 expression in miR-3065-3p-overexpressing HCT116 or RKO cells. **c** Western blot analysis of CRLF1 expression in HCT116 or RKO cells ectopically expressing miR-3065-3p and vector control (MCS) cells. The right panel shows the statistical results. **d** Western blot analysis and statistical results of CRLF1 expression in tumor tissues from the xenograft mouse model. ‘1–4’ represented the different four mice in the same group. **e** Representative images of immunohistochemical staining with antibodies against CRLF1 in tumor tissues. The right panel shows the statistical results. **f** Predicted miR-3065-3p binding sites in the 3ʹUTR of CRLF1 and the seed sequences of miR-3065-3p of different species. **g** The wild-type (WT) or mutant (Mut) construct with miR-3065-3p (accession number: MIMAT0015378) binding sites in the 3ʹUTR of CRLF1. **h** HEK293T cells were cotransfected with a pmirGLO reporter harboring the 3ʹUTR of CRLF1 with wild-type (WT) or mutated (Mut) miR-3065-3p binding sites and miR-3065-3p mimics or NC. Luciferase activity was analyzed 40 h after transfection. **i** Analysis of CRLF1 expression in colorectal cancer and normal tissues according to data from TCGA database using UALCAN website ( http://ualcan.path.uab.edu/index.html). **j**, **k** RT-qPCR (**j**) and western blot (**k**) analysis of CRLF1 expression in colorectal cancer and adjacent normal tissues. **l** Correlation analysis between CRLF1 and miR-3065-3p expression in colorectal cancer. The results are shown as the mean values ± SEM of three independent experiments. *P < 0.05; **P < 0.01; ***P < 0.001

To identify the targets of miR-3065-3p in colorectal cancer cells, two bioinformatics tools, TargetScan (http://www.targetscan.org/) and miRDB (http://mirdb.org/), were used to predict a set of common target genes. Moreover, the downregulated genes (Additional file 2: Table S1) in CRC tissues were screened using the GEPIA (http://gepia.cancer-pku.cn/) database. Based on this analysis, we identified TMEM47, CRLF1 and CLDN11 as potential targets (Fig. 4a). When we analyzed the change in the mRNA level of these genes after overexpression of miR-3065-3p in HCT116 or RKO cells, we found that CRLF1 mRNA significantly decreased, while the mRNA levels of TMEM47 and CLDN11 were not obviously changed (Fig. 4b). We next confirmed that CRLF1 protein expression levels were downregulated in HCT116 and RKO-overexpressing miR-3065-3p cells (Fig. 4c). In addition, western blot analysis and immunohistochemical staining showed a reduction in CRLF1 expression in the tumor tissues of the mouse model (Fig. 4d, e).

More importantly, sequence analysis showed that the miR-3065-3p-binding sequences in the 3ʹUTR of CRLF1 mRNA are highly conserved among numerous diverse species (Fig. 4f). Subsequently, the wild-type and mutant miR-3065-3p-binding sites were cloned into luciferase vectors (Fig. 4g). Luciferase reporter assays showed that overexpression of miR-3065-3p suppressed the luciferase activity of the reporter vector containing the wild-type CRLF1 3ʹUTR fragment but not that containing the mutant (Fig. 4h). Moreover, analysis of the TCGA database showed that CRLF1 expression was reduced in colorectal cancer patients compared to normal subjects (Fig. 4i). It was also confirmed that the mRNA and protein expression of CRLF1 was markedly downregulated in colorectal cancer tissues compared to corresponding adjacent normal tissues (Fig. 4j, k). Correlation analysis showed that the levels of miR-3065-3p in CRC tissues were negatively correlated with CRLF1 expression (Fig. 4l). Collectively, these results reveal that CRLF1 is a direct target of miR-3065-3p and that its expression is downregulated by miR-3065-3p in colorectal cancer.

### CRLF1 suppresses the stemness of CRC cells and attenuates the promotion of stemness induced by miR-3065-3p in vitro

**Fig. 5:**
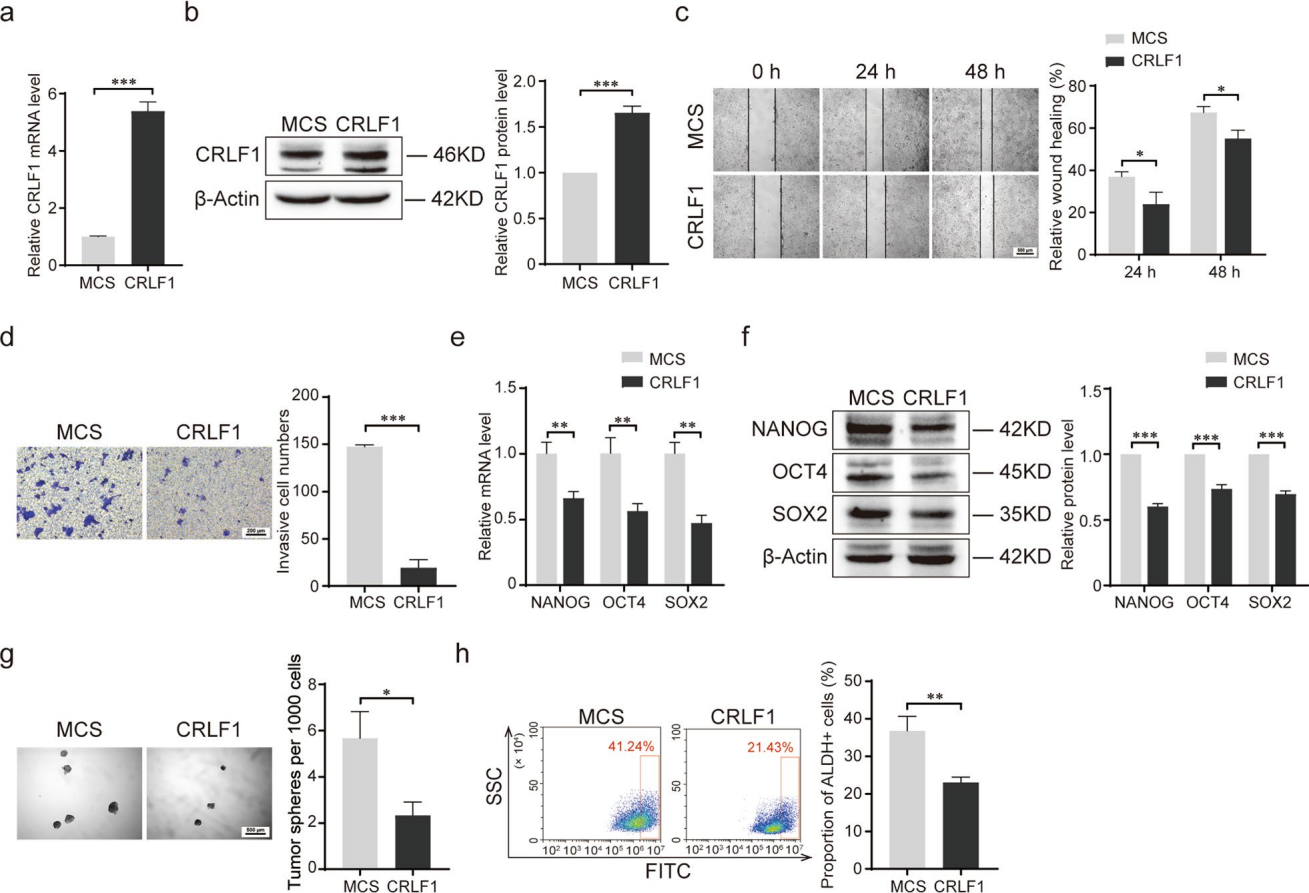
CRLF1 suppresses the stemness of colorectal cancer cells in vitro*.* **a**, **b** RT-qPCR (**a**) and western blot (**b**) analysis of CRLF1 overexpression in HCT116 cells. **c** Representative images of the wound healing assay and statistical results of the migration area are shown. **d** Transwell assay of the migration of CRLF1-overexpressing HCT116 cells and vector controls (MCS)-expressing cells and the statistical results. **e**, **f** The mRNA (**e**) and protein (**f**) levels of NANOG, OCT4, and SOX2 in CRLF1-overexpressing HCT116 cells were analyzed by RT-qPCR and western blotting. **g** Representative images and statistical results of sphere formation assays. **h** Flow cytometric analysis of ALDH activity in CRLF1-overexpressing HCT116 cells. Statistical results are shown in the right panel. The data shown represent the mean values ± SEM of three independent experiments. *P < 0.05; **P < 0.01; ***P < 0.001

Cytokine Receptor Like Factor 1 (CRLF1), a member of the cytokine type I receptor family, forms a secreted complex with cardiotrophin-like cytokine factor 1 and acts on cells expressing ciliary neurotrophic factor receptors. It has been reported that CRLF1 is involved in the regulation of neuronal development and tumor progression. To determine the role of CRLF1 in colorectal cancer, we established a HCT116 colorectal cancer cell line stably overexpressing CRLF1 (Fig. 5a, b). First, cell invasion and migration were assessed by wound healing and transwell assays. Our results revealed that overexpression of CRLF1 significantly reduced the invasion and migration ability of HCT116 cells (Fig. 5c, d). Then, the effect of CRLF1 on the stem cell-like characteristics of colorectal cancer was evaluated. The results showed that overexpression of CRLF1 inhibited the mRNA and protein expression levels of NANOG, OCT4 and SOX2 (Fig. 5e, f). Sphere formation and flow cytometric anlysis showed that overexpression of CRLF1 significantly suppressed sphere formation ability and ALDH activity (Fig. 5g, h). Therefore, these results indicated that CRLF1 functions as a tumor supperssor and inhibits the stemness of colorectal cancer cells.

**Fig. 6:**
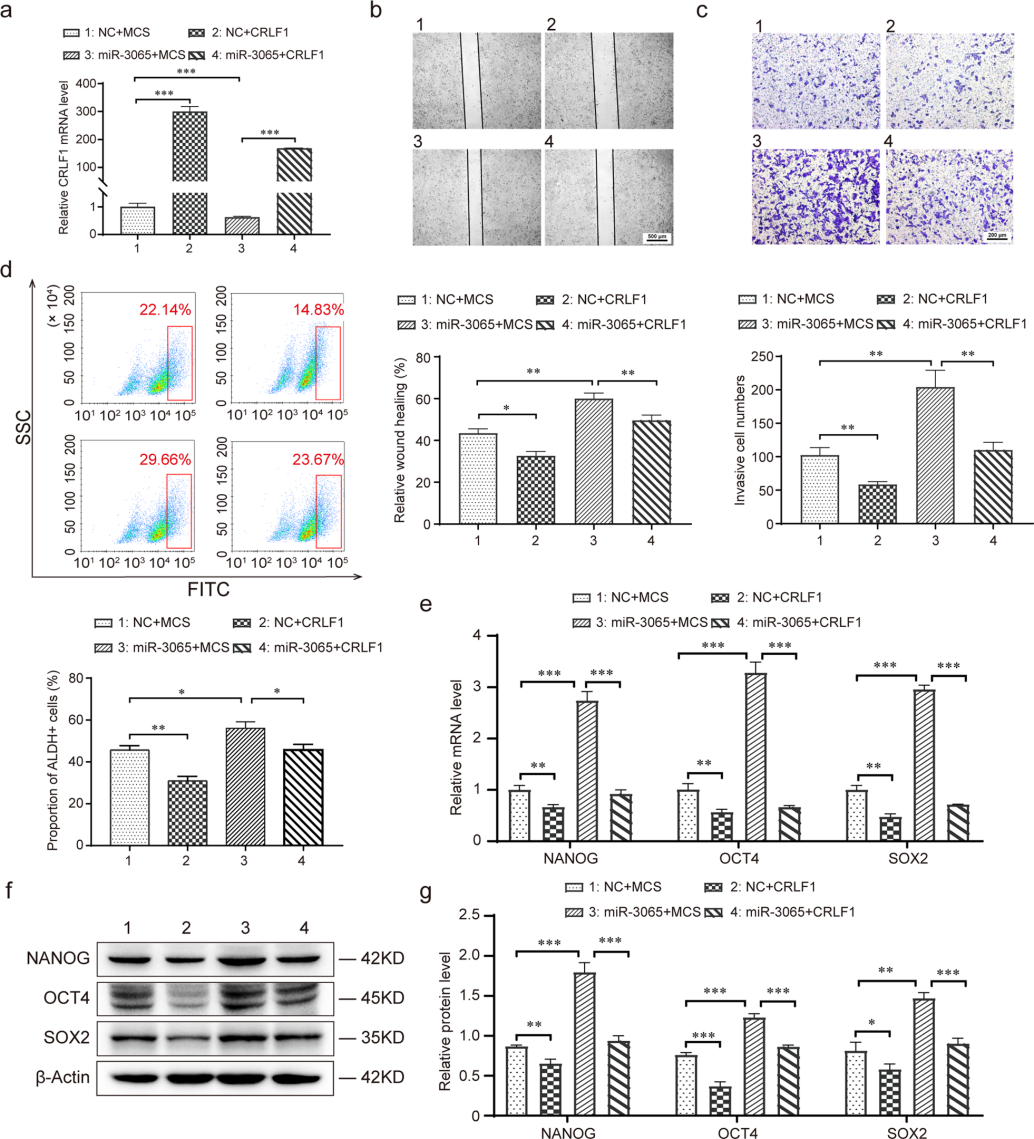
CRLF1 attenuates the promotion effect of stemness induced by miR-3065-3p in CRC cells*.* **a**, **b** miR-3065-3p-overexpressing HCT116 cells or control cells (NC) were transfected with vector control (MCS) or CRLF1 plasmid for 24 h. (**a**) RT-qPCR analysis of CRLF1 expression in HCT116 cells. (**b**) Representative images of the wounding healing assay (up) and statistical results of wound healing area (down) were shown. **c** Transwell assay of migrated cells (up) and statistical results (down) of the indicated groups. **d**–**f** miR-3065-3p-overexpressing HCT116 cells or control cells (NC) were transfected with vector control (MCS) or CRLF1 plasmid for 48 h. **d** Flow cytometric analysis of ALDH activity (up) and its statistical results (down) were shown. RT-qPCR (**e**) and western blot (**f**, **g**) analysis of NANOG, OCT4, and SOX2 expression in HCT116 cells. The data shown represent the mean values ± SEM of three independent experiments. *P < 0.05; **P < 0.01; ***P < 0.001

Furthermore, we explored the role of CRLF1 in the effect of miR-3065-3p on the expression of stemness-related transcription factors. The results showed that overexpression of CRLF1 rescued the increase in the ability of migration and invasion, ALDH activity and stemness-related transcription factor NANOG, OCT4 and SOX2 expression, which were caused by overexpression of miR-3065-3p (Fig. 6a–f). Taken together, these results suggested that CRLF1 mediates the stemness-promoting effect of miR-3065-3p in colorectal cancer cells.

### CRLF1 inhibits the tumorigenesis and metastasis of colorectal cancer in vivo

**Fig. 7:**
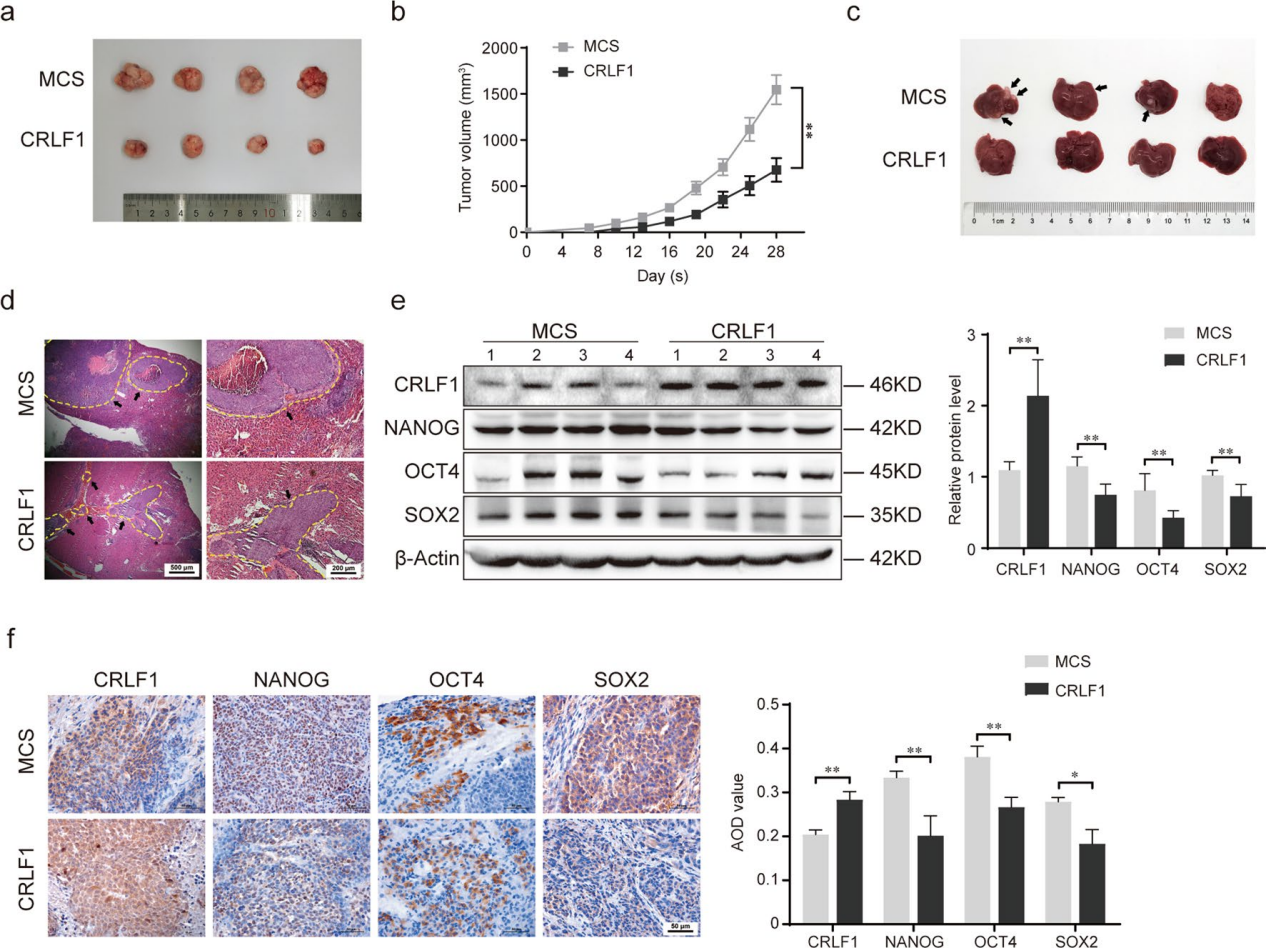
CRLF1 inhibits tumorigenesis and metastasis of colorectal cancer in vivo*.* **a**, **b** A mouse model of colorectal cancer with five mice in each group was established via orthotopic implantation of HCT116 cells overexpressing CRLF1 or vector control (MCS). Images showing the tumor size (**a**) and statistical results of tumor volume (**b**) are shown. A liver metastasis mouse model of colorectal cancer with five mice in each group was established via spleen injection using CRLF1-overexpressing HCT116 or control cells. **c**, **d** Metastatic nodules of the liver (**c**) and representative images of HE-stained metastatic lesions by (**d**) are shown. **e**,** f** Western blot (**e**) and immunohistochemical (**f**) analysis of NANOG, OCT4, and SOX2 expression in tumor tissue from xenograft mice. ‘1–4’ represented the different four mice in the same group. Statistical results are shown in the right panel. The data shown represent the mean values ± SEM of three independent experiments. *P < 0.05; **P < 0.01; ***P < 0.001

To investigate the impact of CRLF1 on the tumorigenesis and metastasis of colorectal cancer, we established a xenograft tumor model and liver metastasis model of colorectal cancer using HCT116-overexpressing CRLF1 or control cells. The results showed that in the xenograft models, CRLF1 overexpression significantly suppressed the growth of tumors (Fig. 7a, b). Consistently, CRLF1 significantly inhibited the formation of hepatic metastatic nodules in the liver metastatic model (Fig. 7c, d). Furthermore, we also measured the expression of stemness-related transcription factors in tumor tissues by western blot analysis and immunohistochemistry. As expected, the expression of NANOG, OCT4 and SOX2 was decreased in mice ectopically expressing CLRF1 compared to controls (Fig. 7e, f). Collectively, these results demonstrated that CRLF1 inhibits the tumorigenesis and metastasis of colorectal cancer in vivo.

## Discussion

Colorectal cancer (CRC) is one of the most common primary malignancies of the digestive tract and is a serious threat to public health. Although CRC therapies have significantly been improved, the prognosis of patients with CRC is still poor. Cancer stem cells (CSCs) are thought to be the root of tumor initiation, progression, metastasis, and therapeutic resistance, which have been confirmed in numerous tumor-bearing mouse models [31]. Therefore, there is an urgent need to identify novel therapeutic targets and identify the regulatory mechanism of colorectal cancer cell stemness.

In this study, we found that miR-3065-3p expression increased significantly in CRC tissues using the TCGA dataset analysis. It was also confirmed by 17 cases of clinical samples, which is also a defect of this study due to the insufficient clinical sample size. Therefore, it is necessary to expand the sample size for further validation in the future. In addition, high expression of miR-3065-3p was associated with poor prognosis of patients with CRC. More importantly, overexpression of miR-3065-3p promoted the stem cell-like characteristics of CRC cells in vitro and in vivo. Consistent with our findings, miR-3065 has been shown to interact with LINC01133 in the ceRNA network and to play a key role in the occurrence and development of cervical cancer at different stages [18]. However, several studies have reported that miR-3065-3p serves as a tumor suppressor. For example, downregulation of miR-3065-3p expression is associated with poor prognosis in several cancer cases with p53 mutated, including gastric cancer, breast cancer and liver cancer cases [17]. In addition, miR-3065-5p functions as an antitumor miRNA to inhibit the proliferation of melanoma cells by targeting the HIPK1 and ITGA1 genes [32]. This suggests that miR-3065-3p plays different roles depending on the cancer type.

Evidence has indicated the potential diagnostic and prognostic role of circulating miRNAs or serum factors. For example, extracellular vesicle-miR-101 is a promising circulation biomarker for metastasis of osteosarcoma [33]. Habib et al*.* reported that circulating miR-146a expression can predict the early response to imatinib treatment in patients with chronic myeloid leukemia [34]. And, Muhammad et al. reported that serum Metadherin mRNA expression can be used for screening and early diagnosis of CRC [35]. In addition, serum neuropilin-1 and angiopoietin-2 are potential markers for hepatocellular carcinoma diagnosis [36]. Similarly, serum high-temperature-required protein A2 (HtrA2) was reported as a potential biomarker for the diagnosis of breast cancer [37]. In this study, we found that high levels of miR-3065-3p positively correlated with poor prognosis in CRC, suggesting that miR-3065-3p may serve as a diagnostic and prognostic marker of CRC, and future extracellular diagnostic studies will be conducted.

Furthermore, we demonstrated that CRLF1*,* a member of the cytokine receptor family, was the target of miR-3065-3p. It has been reported that CRLF1 promotes the proliferation and survival of normal neuronal cells and B-cells by binding to cardiotrophin-like cytokine factor 1 (CLCF1), a member of the IL-6 family of cytokines [38]. More importantly, CRLF1 is involved in the regulation of tumor progression. For example, the expression level of CRLF1 is increased in lung adenocarcinoma tissues compared to normal tissues and promotes malignant phenotypes of papillary thyroid carcinoma by activating the MAPK/ERK and PI3K/AKT pathways [39–41]. in addition, CRLF1 is considered as a potential target gene of a tumor suppressor TNRC6C in papillary thyroid cancer [42]. Here, we showed that CRLF1 expression was decreased in CRC and that overexpression of CRLF1 inhibited the stemness and metastasis of CRC cells in vitro and in vivo. Moreover, ectopic expression of CRLF1 attenuated the promoting effect of miR-3065-3p on the stemness of CRC cells. These studies suggest that CRLF1 plays a tumor suppressor role in colorectal cancer progression. However, the regulatory mechanism of CRLF1 in stemness inhibition needs to be further explored.

In summary, we demonstrated that miR-3065-3p was overexpressed in colorectal cancer and its expression was associated with poor prognosis in patients with colorectal cancer. miR-3065-3p significantly promoted stemness and liver metastasis in vitro and in vivo. Furthermore, we found that CRLF1 was the direct target of miR-3065-3p and functioned as a suppressor of the stemness of colorectal cancer cells. Moreover, CRLF1 mediated the promoting effect of miR-3065-3p on stemness-related transcription factor expression in colorectal cancer cells. Our present findings indicated that miR-3065-3p might serve as a prognostic marker as well as a therapeutic target for colorectal cancer.

## Conclusions

**Fig. 8:**
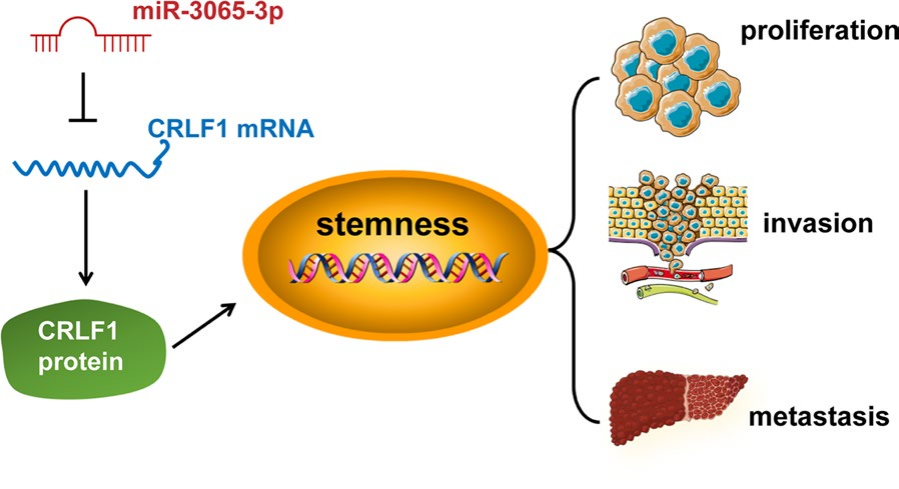
A schematic diagram illustrating the role of miR-3065-3p/CRLF1 in promoting colorectal cancer metastasis and stemness. miR-3065-3p promotes the promotes metastasis and stemness and by targeting CRLF1 in colorectal cancer

In conclusion, our study demonstrated that miR-3065-3p was overexpressed in CRC and its high expression was associated with poor prognosis in patients with CRC. More importantly, miR-3065-3p promoted stemness and liver metastasis of CRC by inhibition of downstream gene *CRLF1* (Fig. 8). Our finding indicates that miR-3065-3p represents a promising prognostic marker and target for the treatment of colorectal cancer.

## Supplementary Information

**Additional file 1:** Figure S1

**Additional file 2:** Table S1

## Data Availability

The datasets analyzed in this study are available from the corresponding author on reasonable request.

## Abbreviations

CRC: Colorectal cancer
CSC: Cancer stem cells
miRNA: MicroRNA
CRLF1: Cytokine receptor-like factor 1
TCGA: The Cancer Genome Atlas
DMEM: Dulbecco’s modified Eagle’s medium
FBS: Fetal bovine serum
NC: Negative control
UTR: Untranslated regions
WT: Wild-type
Mut: Mutant
HE: Hematoxylin–eosin
IHC: Immunohistochemistry
