# Supplementary material for: miR-3065-3p promotes stemness and metastasis by targeting CRLF1 in colorectal cancer
Source: J Transl Med. 2021 Oct 16;19:429. doi: 10.1186/s12967-021-03102-y (PMC8520297; doi:10.1186/s12967-021-03102-y)
Supplement: Supplementary file 1 — Additional file 1: Figure S1 [file 12967_2021_3102_MOESM1_ESM.docx]

**
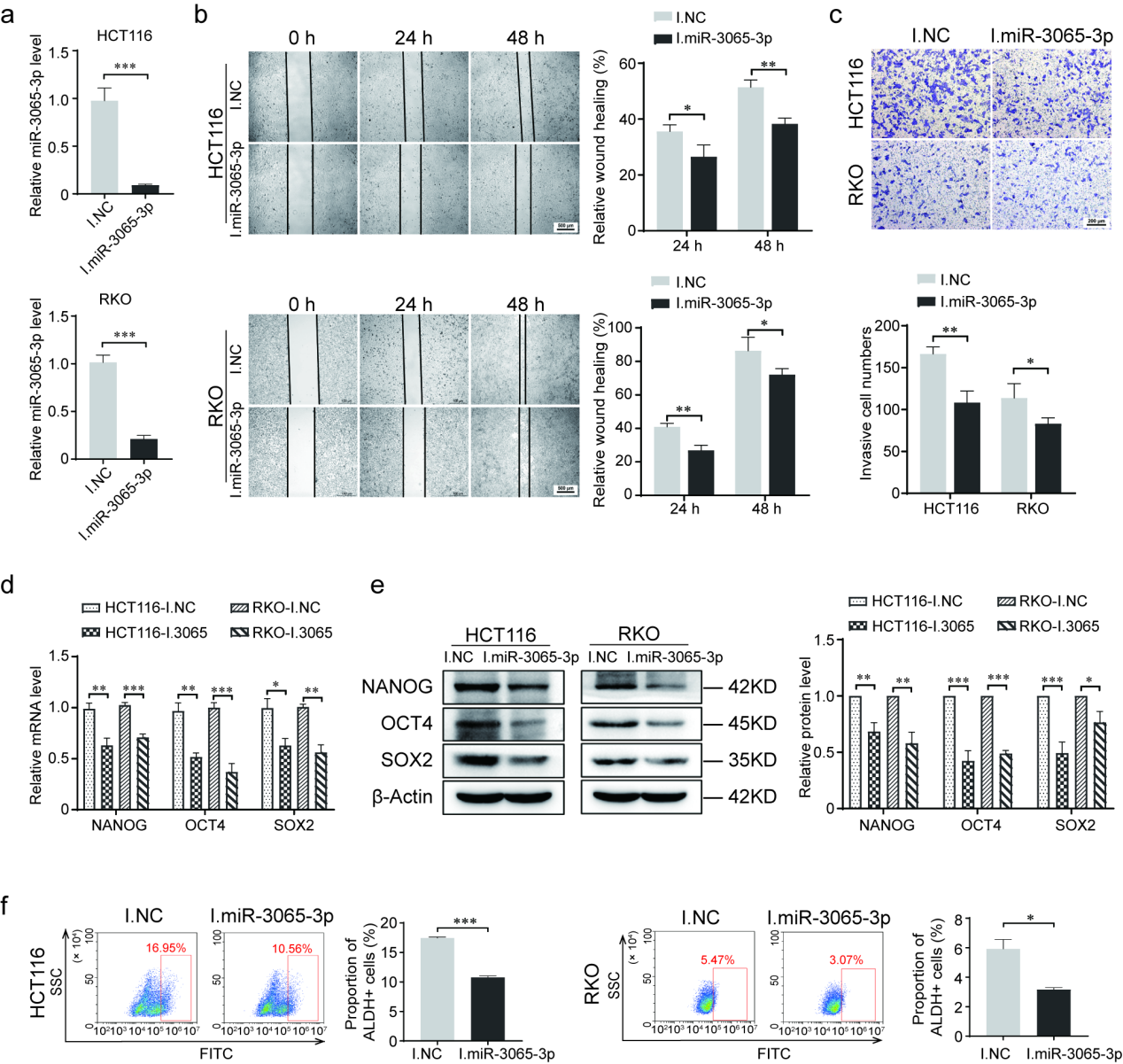
**

**Additional file 1: Figure S1. The low expression of miR-3065-3p inhibits the stemness of colorectal cancer cells *in vitro*.** **a** RT-qPCR analysis of miR-3065-3p inhibitor transfection efficiency in HCT116 and RKO cells. **b** Representative images of the wound healing assay and statistical results of the migration area are shown. **c** Transwell assay of the migration of HCT116 or RKO cells with lower miR-3065-3p expression (I.miR-3065-3p) compared and negative control (I.NC) -expressing cells and the statistical results. **d, e** The mRNA (**d**) and protein (**e**) levels of NANOG, OCT4, and SOX2 in HCT116 or RKO cells with miR-3065-3p down-regulated were analyzed by RT-qPCR and western blotting. **f** Flow cytometric analysis of ALDH activity in HCT116 and RKO cells transfected with miR-3065-3p inhibitor or negative control. Statistical results are shown in the right panel. The data shown represent the mean values ± SEM of three independent experiments. **P* < 0.05; ***P* < 0.01; ****P* < 0.001.
