# Supplementary material for: miR-3065-3p promotes stemness and metastasis by targeting CRLF1 in colorectal cancer
Source: J Transl Med. 2021 Oct 16;19:429. doi: 10.1186/s12967-021-03102-y (PMC8520297; doi:10.1186/s12967-021-03102-y)
Supplement: Supplementary file 2 — Additional file 2: Table S1 [file 12967_2021_3102_MOESM2_ESM.docx]

| **number** | **Downregulated genes** |
| --- | --- |
| 1 | CRLF1 |
| 2 | CLDN11 |
| 3 | TMEM47 |
| 4 | RIMKLB |
| 5 | FAM65A |
| 6 | RP11-834C11.7 |
| 7 | CCBE1 |
| 8 | ZNF248 |
| 9 | AGAP9 |
| 10 | TPM2 |
| 11 | BGLAP |
| 12 | CCDC154 |
| 13 | RGS5 |
| 14 | SFRP2 |
| 15 | BVES-AS1 |
| 16 | ANKRD6 |
| 17 | CAPRIN2 |
| 18 | INSC |
| 19 | STX1B |
| 20 | EGFL8 |
| 21 | AC108488.4 |
| 22 | NUDT10 |
| 23 | REEP2 |
| 24 | SMARCD3 |
| 25 | CTF1 |
| 26 | TMEM59L |
| 27 | ALOX12-AS1 |
| 28 | C1QTNF4 |
| 29 | PTRF |
| 30 | CALB2 |
| 31 | ENO3 |
| 32 | LINC00702 |
| 33 | CCDC136 |
| 34 | CPEB1 |
| 35 | RP11-235E17.6 |
| 36 | SCG2 |
| 37 | MGP |
| 38 | AC007326.1 |
| 39 | HDGFRP3 |
| 40 | HSPB2 |
| 41 | AKAP12 |
| 42 | SPOCK1 |
| 43 | NGF |
| 44 | AHNAK2 |
| 45 | SPAG8 |
| 46 | CRYAB |
| 47 | CTC-524C5.2 |
| 48 | C3orf18 |
| 49 | RASA4B |
| 50 | FSTL3 |
| 51 | MYL9 |
| 52 | RYR2 |
| 53 | CYP2E1 |
| 54 | LPPR2 |
| 55 | BEST2 |
| 56 | SCRG1 |
| 57 | BAALC |
| 58 | HSPA1A |
| 59 | CPT1C |
| 60 | CRIP2 |
| 61 | CNN1 |
| 62 | WDR86 |
| 63 | ARHGEF25 |
| 64 | CSDC2 |
| 65 | TMEM91 |
| 66 | PCOLCE2 |
| 67 | RP11-504P24.3 |
| 68 | ENO2 |
| 69 | LDLRAD2 |
| 70 | MMP23B |
| 71 | C2orf74 |
| 72 | INAFM1 |
| 73 | PRSS53 |
| 74 | FAM223A |

**Additional file 2: Table S1. The downregulated genes in CRC tissues were screened using the GEPIA (http://gepia.cancer-pku.cn/) database.**
